# Effects of Liner-Bonding of Implant-Supported Glass–Ceramic Crown to Zirconia Abutment on Bond Strength and Fracture Resistance

**DOI:** 10.3390/ma12172798

**Published:** 2019-08-30

**Authors:** Yong-Seok Jang, Sang-Hoon Oh, Won-Suck Oh, Min-Ho Lee, Jung-Jin Lee, Tae-Sung Bae

**Affiliations:** 1 Department of Dental Biomaterials, Institute of Biodegradable Materials, BK21 plus Program, School of Dentistry, Chonbuk National University, Jeonju 54896, Korea; 2 Haruan Dental Clinic, Department of Dental Biomaterials, Institute of Biodegradable Materials, BK21 plus Program, School of Dentistry, Chonbuk National University, Jeonju 54896, Korea; 3 Department of Biologic and Materials Sciences Division of Prosthodontics, School of Dentistry, University of Michigan, Ann Arbor, MI 48109, USA; 4 Department of Prosthodontics, Institute of Oral Bio-Science, School of Dentistry, Chonbuk National University and Research Institute of Clinical Medicine of Chonbuk National University-Biomedical Research Institute of Chonbuk National University Hospital, Jeonju 54907, Korea

**Keywords:** CAD/CAM all-ceramic restoration, fracture strength, liner treatment, resin cement, tensile bond strength, zirconia abutment

## Abstract

This study was conducted to test the hypothesis that heat-bonding with a liner positively affects the bond strength and fracture resistance of an implant-supported glass–ceramic crown bonded to a zirconia abutment produced by a computer-aided design/computer-aided milling (CAD/CAM) procedure. Lithium disilicate-reinforced Amber Mill-Q glass ceramic blocks were bonded to 3 mol% yttria stabilized tetragonal zirconia polycrystal (3Y-TZP) blocks by heat-bonding with a liner or cementation with a dual-cure self-adhesive resin cement for a microtensile bond strength test. CAD/CAM implant-supported glass ceramic crowns were produced using Amber Mill-Q blocks and bonded to a milled 3Y-TZP zirconia abutments by heat-bonding or cementation for a fracture test. A statistical analysis was conducted to investigate the significant differences between the experimental results. The mode of failure was analyzed using high-resolution field emission scanning electron microscopy. Chemical bonding was identified at the interface between the zirconia ceramic and liner. The mean tensile bond strength of the liner-bonded group was significantly higher than that of the cement-bonded group. The initial chipping strength of the liner-bonded group was significantly higher than that of the cement-bonded group, although no statistically significant difference was found for the fracture strength. The mode of failure was mixed with cohesive fracture through the liner, whereas the cement-bonded group demonstrated adhesive failure at the interface of bonding.

## 1. Introduction

Implant-supported crowns restore oral functions and aesthetics without affecting the integrity of adjacent teeth. These crowns are usually connected to the implants by means of titanium abutments. However, the use of a metallic abutment may compromise the gingival aesthetics of implant crowns, particularly in patients presenting with a thin biotype gingival architecture. The grayish metallic color can be pronounced with a light reflected from the metallic surface of the abutment [1,2]. In addition, the submucosal placement of a crown margin can restrict the cement removal procedure and lead to develop peri-implantitis [3].

Ceramic abutments are biocompatible and aesthetic by mimicking the color of natural teeth [4,5]. In addition, the use of ceramic abutments is versatile with the development of CAD (computer-aided design)/CAM (computer-aided milling) technology, which allows for the design and milling of zirconia ceramics. Though concerns regarding the mechanical properties of zirconia abutments (i.e., brittleness, stress corrosion cracking, and low temperature degradation) have been addressed, their clinical application has been expanded for the anterior and premolar regions [6,7,8,9]. Furthermore, all abutments made of zirconia or titanium can be customized to produce a desired size, shape, angle, and location of a crown margin for better aesthetics [10,11,12].

Zirconia abutments are commonly designed as one-piece system to support implant crowns and establish the implant–abutment interface with a zirconia ceramic. This system eliminates the negative effects of titanium abutments related to aesthetics. However, the implant–abutment interface may experience an excessive wear under occlusal loading due to dissimilarities between the zirconia ceramic and the titanium implant [13,14]. Complications may include a discoloration of the peri-implant mucosa associated with the embedment of titanium particles dislodged from implants, abutment screw loosening, and the fracture of ceramic abutments and crowns [14,15]. This type of abutments have also shown very low fracture resistance when compared to zirconia abutments using a titanium base [16,17].

The two-piece system of zirconia abutments was designed to avoid the possible consequences related to the use of the one-piece system. This system consists of a ceramic core and a titanium link to establish the implant–abutment interface with titanium-to-titanium without compromising the aesthetics of the ceramic abutment. The ceramic core is customized using CAD/CAM technology and is bonded to the titanium base with adhesive resin cement [18]. This two-piece system was found to demonstrate a higher flexural resistance than one-piece system [1,19] without compromising the emergence profile, crown orientation, and coronal contour matching the prosthesis and anatomical shape of the mucosa [20].

However, problems may occur when bonding a final prosthesis to a zirconia ceramic abutment [18,21,22]. The resin cement may not adhere to the zirconia ceramic abutment because of the lack of undercut features commonly created by blasting with air-borne particle abrasion technology and/or etching with hydrofluoric acid. The final prosthesis can be at a risk of chipping or cracking due to the relatively weak bond strength of the resin cementing the abutment. In addition, the luting procedure of the final prosthesis to the abutment is inconvenient and can be problematic when combined with the submucosal margin.

A lithium disilicate-reinforced liner has recently been developed to overcome the weak linkage of the final prosthesis of a glass–ceramic to a zirconia abutment designed to replace single anterior and posterior teeth. However, no scientific research has been conducted to elucidate the bond characteristics of the liner. The objective of this in vitro study was to investigate the effect of heat-bonding with the liner on the bond strength and fracture resistance of an implant-supported glass–ceramic crown bonded to a two-piece system zirconia abutment produced by a CAD/CAM procedure. The null hypothesis was set to test no significant difference between the liner-bonding and the cement-bonding of the implant-supported ceramic crown to the zirconia abutment on the initial chipping and fracture strengths of crowns, as well as the microtensile bond strength.

## 2. Materials and Methods

### 2.1. Bond Strength Test

#### 2.1.1. Preparation of Specimen

Three CAD/CAM 3Y-TZP zirconia ceramic blocks (Zirtooth, O98FGJ1701, Hass, Gangneung, Korea) were machined to fabricate 6 zirconia ceramic specimens with dimensions of 10 × 10 × 5 mm, and they were sintered in an electric furnace (Programat EP3000/G2, Ivoclar Vivadent, Schaan, Liechtenstein) at 1450 °C. In addition, lithium disilicate-reinforced Amber Mill-Q glass ceramic blocks (Hass, Gangneung, Korea) were machined to fabricate 6 glass ceramic specimens matching the zirconia ceramic specimens (10 mm × 10 mm × 5 mm).

For bonding with the liner, the liner (Hass, Gangneung, Korea), consisting of 5–15 wt.% Li_2_O, 55–65 wt.% SiO_2_, and 5–25 wt.% other trace elements of oxides and colorants was applied on the surfaces of 3 zirconia ceramic specimens. Then, heat-bonding with 3 lithium disilicate-reinforced glass ceramic specimens was conducted at 800 °C. 

For bonding with a resin cement, 3 lithium disilicate-reinforced glass ceramic specimens were acid-etched with 9.5% hydrofluoric acid gel (Bisco, IL, USA) for 30 s, washed with distilled water, dried, and silane primer (Espe^TM^ Sil, 3M/ESPE, Seefeld, Germany) coated. The surfaces of 3 zirconia ceramic specimens were roughened using an air-borne particle abrasion technology with 50 µm alumina particles (Hi-aluminas, Shofu, Japan) under the pressure of 3 atm at a distance of 10 mm [23]. After placing the specimens in an electric furnace (Programat EP3000/G2), the temperature was raised to 1000 °C at a rate of 50 °C/min, held for 10 min, and then cooled to 25 °C in the furnace to restore the phase transformations occurred during the blasting procedure. Then, a 10-methacryloyloxydecyl dihydrogen phosphate (MDP)-containing primer (Z-PRIME^TM^ plus, BISCO, Schaumburg, IL, USA) was coated on the surface of zirconia ceramic specimens [24]. The cement bonding between the lithium disilicate-reinforced glass and zirconia ceramics was performed using a self-adhesive dual-cure resin cement (Rely-X^TM^ U200, 3M/ESPE, Neuss, Germany) with an equal amount of base and catalyst pastes mixed for 20 s. The cement was applied to the prepared ceramic surface and a pressure of 49 N was applied to the ceramic specimens under a constant-load device. After the removal of excess cement, the resin cement was photopolymerized using an light emitting diode (LED) curing unit (G-Light, GC Corporation, Tokyo, Japan) for 20 s from each of the four directions for a total of 80 sec under a light intensity of 550 mW/cm^2^ at a distance of 2 mm. The specimens were kept under pressure for additional 10 min, relieved from the constant-load device and immersed in distilled water for 24 h.

**Figure 1:**
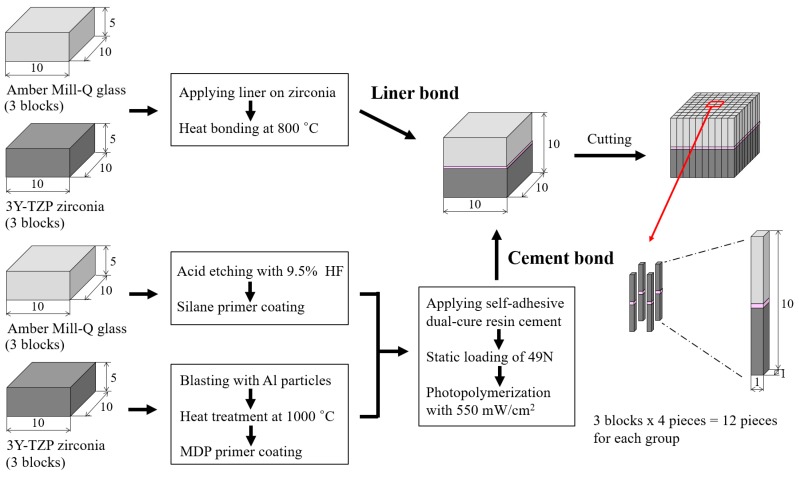
Schematic summary for the preparation process of specimens for the microtensile bond test.

The bonded ceramic blocks were serially sectioned perpendicular to the bonded surface. The first cut was made through each specimen using a high-speed diamond cutting machine (Accutom-50, Struers Inc, Cleveland, OH, USA) to produce a 1 mm thick plate, and the sliced plate was cut using a low-speed diamond cutting machine (Metsaw-LS, Topmet, Daejeon, Korea) after rotating 90° for second set of cuts. Twelve specimens (1.0 mm × 1.0 mm × 10 mm) were prepared with 3 ceramic blocks per each group by selecting 4 specimens from the middle of a bonded ceramic block (10 mm × 10 mm × 10 mm). Thus, a total of 24 specimens (12 × 2 groups) were used for the bond strength test. Figure 1 schematically shows the preparation process of specimens for the microtensile bond test.

#### 2.1.2. Microtensile Bond Strength Test

The prepared ceramic specimens were attached to the grip of metal holders, mounted, and subjected to tensile force in a universal testing machine (Instron, Model 5569, Instron Co., Norwood, MA, USA) at a crosshead speed of 0.5 mm/min [25,26]. The microtensile bond strength was calculated in MPa with the failure load (N) divided by the cross-sectional area (mm^2^) of each test specimen.

### 2.2. Fracture Test for Implant-Supported Crowns

#### 2.2.1. Preparation of Implant-Supported Crowns

The mandibular right first molar was scanned using a D900L scanner (3Shape, Copenhagen, Denmark) to create an implant-supported crown. Twenty ceramic crowns were produced by milling a lithium disilicate-reinforced glass ceramic block using CAD/CAM milling machine (CEREC MC, Sirona, Salzburg, Austria) to make 10 samples per group for the liner-bonded and cement-bonded groups. The internal aspect of each crown was acid-etched for bonding to a zirconia abutment presenting with a diameter of 4 mm and length of 8 mm. The bonding was performed using the same method as described in the section of the bond strength test for each liner-bonded and cement-bonded group. The zirconia abutments were connected to the titanium link abutments (SRN-SURO-H, GeoMedi Co, Ltd., Uiwang, Gyeonggi, Korea) with a dual-cure resin cement after applying an MDP containing primer. 

The prepared crowns were coated with a thin layer of e.max Ceram glaze paste (Ivoclar/Vivadent, Schaan, Liechtenstein) and glazed in an electric furnace (Programat EP3000/G2). The temperature was raised to 730 °C at a rate of 35 °C/min and held for 1 min. For the liner-bonded group (MLG: Milled, liner-bonded and glazed), the ceramic crowns were glazed following the bonding with the zirconia abutments. However, for the cement-bonded group (MGC: Milled, glazed, and cement-bonded), the ceramic crowns were glazed prior to bonding to the abutments.

#### 2.2.2. Fracture Test of Implant-Supported Crown

The prepared ceramic crown/abutment specimens were connected to titanium implant analogs (Geo 3D Analog, GeoMedi Co, Ltd., Uiwang, Gyeonggi, Korea) with titanium abutment screws and tightened to 30 N·cm twice at 30 seconds interval using a torque gauge (9810P, Aikoh Engineering Co, Higashiosaka, Osaka, Japan) [27]. The screw access holes were filled with a light-activated composite resin (Filteck^TM^ Z350 XT, 3M ESPE, MN, USA), and the prepared specimens were immersed in distilled water at 37 °C for 24 h. 

The crowns were supported and secured to a base retainer and loaded axially to fracture in a universal testing machine (Universal testing machine 4201, Instron, Canton, MA, USA). The compression load was transferred through a 5 mm diameter steel ball positioned in the central fossa of the crown. The load was applied at a crosshead speed of 0.5 mm/min and continued to the point of fracture, and the fracture load of each ceramic crown was recorded [28,29]. The initial chipping strength was determined at the point when the load value showed slightly transient drop. 

### 2.3. Surface Analysis

High-resolution field emission scanning electron microscopy (HR FE-SEM in KBSI Jeonju, SU8230, Hitachi, Japan) was used to investigate the topography of the liner-bonded surface, cross-sectional bonded interfaces, and fractured surfaces after etching with a 9.5% hydrofluoric acid gel for 30 s. The liner layer was removed with a 5% hydrofluoric acid solution for 30 min to identify the effects of liner treatment on zirconia ceramic. The distribution of the chemical elements at the bonded interface was analyzed using an energy dispersive X-ray spectrometer (EDS, Bruker, Germany), and the crystal structure on the liner-bonded surface was investigated by X-ray diffraction (XRD, Dmax III-A type, Rigaku, Japan).

### 2.4. Statistical Analysis

The statistical analysis was conducted by SPSS software (version 12.0, SPSS, Chicago, IL, USA). A Student’s t-test was conducted to investigate significant differences between the experimental results for the 2 test groups (*p* < 0.05). A Weibull analysis was performed with the experimentally measured load-at-failure values for each group of bonded ceramic specimens.

## 3. Results

**Figure 2:**
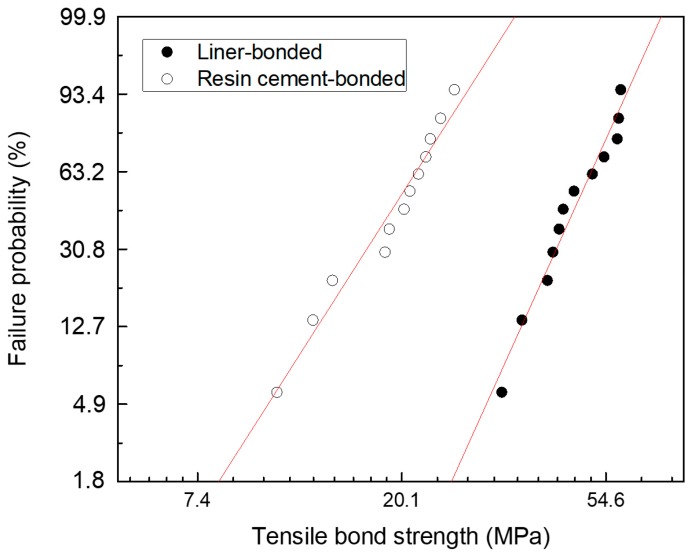
Weibull plots of a microtensile bond strength test for lithium disilicate-reinforced glass ceramic and zirconia of the liner-bonded group and the cement-bonded group.

The null hypothesis on the microtensile bond strength was rejected. Table 1 summarizes the distribution characteristics of the Weibull analysis for the microtensile bond strength test. The Weibull modulus (m) was higher for the liner-bonded group (5.8) than for the cement-bonded group (4.1). The mean values of microtensile bond strength for the liner-bonded group (47.7 MPa) were significantly higher than for the cement-bonded group (19.6 MPa) (*p* < 0.05). In addition, the Weibull distribution showed a matched tendency with a single mode (r^2^ > 0.958) (Figure 2). 

**Figure 3:**
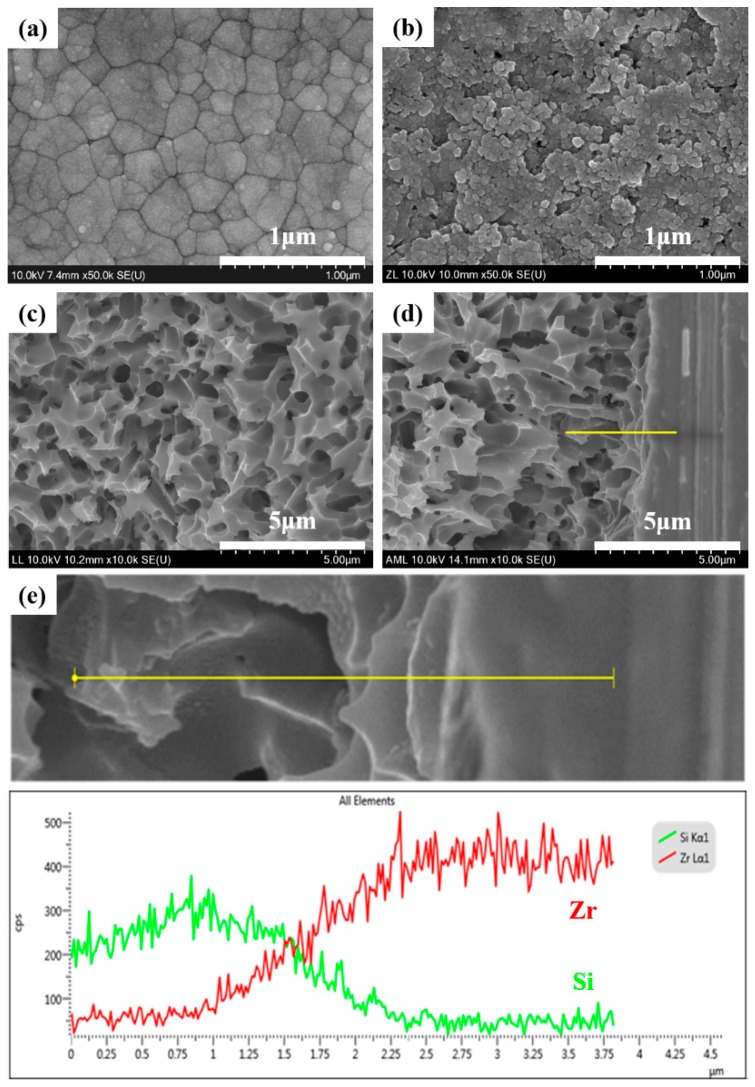
High-resolution field emission scanning electron microscopy (HR FE-SEM) images of the zirconia ceramic specimen demonstrating (**a**) crystal structure after sintering at 1450 °C, (**b**) its alteration of surface morphology with liner treatment (liner removed with acid etching with a 5% hydrofluoric acid solution for 30 min after a fracture test), (**c**) liner-bonded zirconia surface, and (**d**) cross-sectional view of liner-bonded interfacial interaction zone, and (**e**) energy dispersive X-ray spectrometer (EDS) line analysis data of Si and Zr after acid etching with 9.5% hydrofluoric acid gel for 30 s.

The heat-bonding with the liner was found to induce a chemical interaction with the milled zirconia ceramic abutment. A large number of pores was found in the reaction layer of liner bonding, altering the surface topography of the zirconia ceramic (Figure 3a,b). The zone of the chemical reaction was measured approximately 3 μm across the interface of the bonding of the zirconia ceramic (Figure 3d,e).

**Figure 4:**
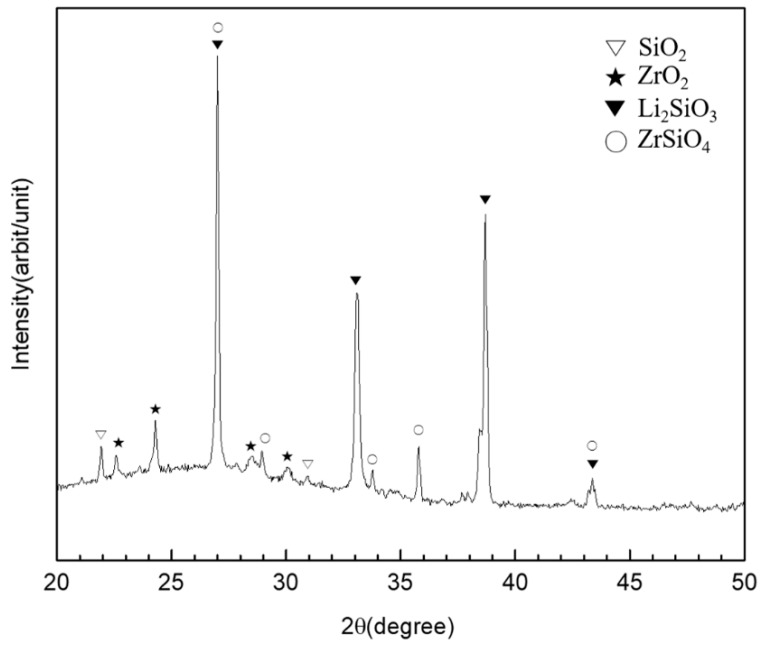
XRD diffraction analysis of the lithium disilicate-reinforced liner-bonded interface of the zirconia ceramic.

The chemical reaction of the liner against the zirconia abutment was confirmed by XRD diffraction analysis, where the peaks were noted as corresponding to lithium metasilicate (Li_2_SiO_3_) and zirconium silicate (ZrSiO_4_), as well as zirconia (ZrO_2_) and silica (SiO_2_) (Figure 4).

**Figure 5:**
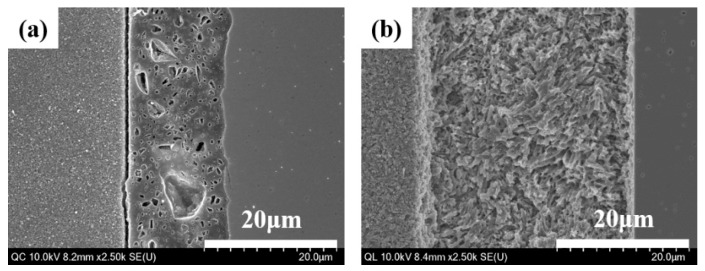
HR FE-SEM images of bonded interface between lithium disilicate-reinforced glass ceramic and zirconia for (**a**) the cement-bonded group and (**b**) the liner-bonded group.

The interfacial layer created by the liner-bonding was found to be more consistent than that created by the cement-bonding. The liner-bonded interfacial layer was thicker with a higher mean value and standard deviation (33.6 ± 5.2 µm) than the cement-bonded interfacial layer (13.3 ± 1.6 µm) (Figure 5). Neither a pore nor a gap was noted in the liner-bonded interfacial zone, whereas numerous micro-pores and micor-gaps were found in the cement-bonded interfacial layer. 

**Figure 6:**
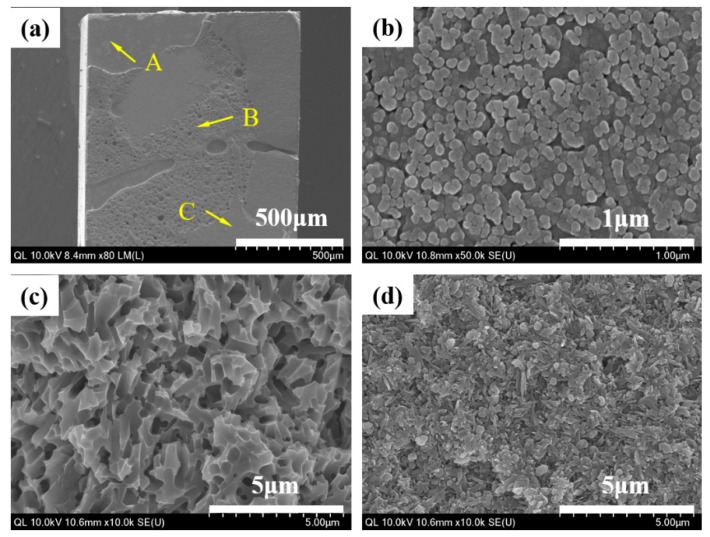
HR FE-SEM images of the liner-bonded group demonstrating mode of failure with the microtensile bond strength test. (**a**) Fracture surface of specimen demonstrating inhomogeneous pattern of fracture, (**b**) magnification of point A demonstrating that the zirconia surface layer reacted with the liner, (**c**) the magnification of point B demonstrating the microstructure of the liner, and (**d**) the magnification of point C demonstrating the presence of needle-shaped lithium disilicate crystals in the lithium disilicate-reinforced glass ceramic.

**Figure 7:**
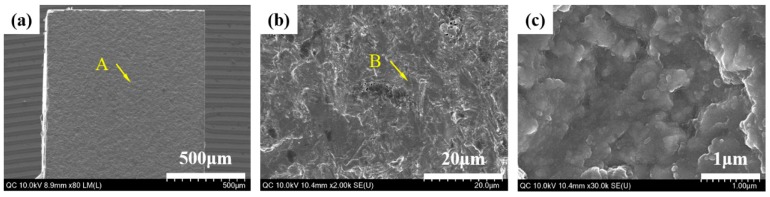
HR FE-SEM images of the cement-bonded group demonstrating mode of failure with the microtensile bond strength test. (**a**) Fractured surface of specimen demonstrating the homogeneous pattern of fracture, (**b**) the magnification of point A demonstrating the irregular structure of the zirconia ceramic, and (**c**) the magnification of point B demonstrating the crystal structure of zirconia.

When the debonded surfaces of the liner-bonded and liner-bonded groups were visually evaluated with high magnification images of HR FE-SEM, the mode of failure of the liner-bonded group was mixed with cohesive fracture propagated through the liner (Figure 6). In contrast, the mode of failure of the cement-bonded group was adhesive where the fracture occurred at the interface between the zirconia ceramic and the cement layer (Figure 7).

**Figure 8:**
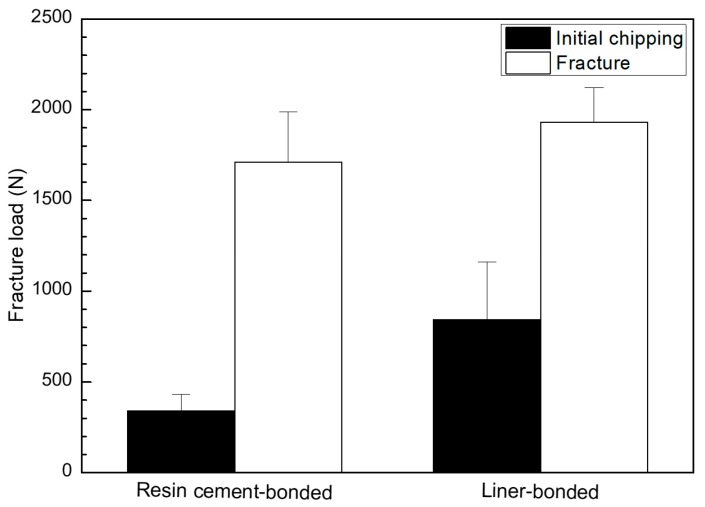
Graphical illustration for initial chipping and fracture strengths of crowns for the resin cement-bonded and liner-bonded groups.

The null hypothesis was rejected on the initial chipping and fracture strengths but accepted on the fracture strength. The mean values and standard deviations of the initial chipping strength and fracture strength of implant-supported glass ceramic crowns bonded to zirconia ceramic abutments were 843.8 ± 317.5 N and 1929.6 ± 191.1 N for the liner-bonded (MLG) groups and 341.0 ± 90.2 N and 1711.1 ± 275.4 N for the resin cement-bonded (MGC) group (Figure 8). The initial chipping strength of the MLG group was significantly higher than that of the MGC group (*p* < 0.05), although no significant difference was found in the fracture strength.

**Figure 9:**
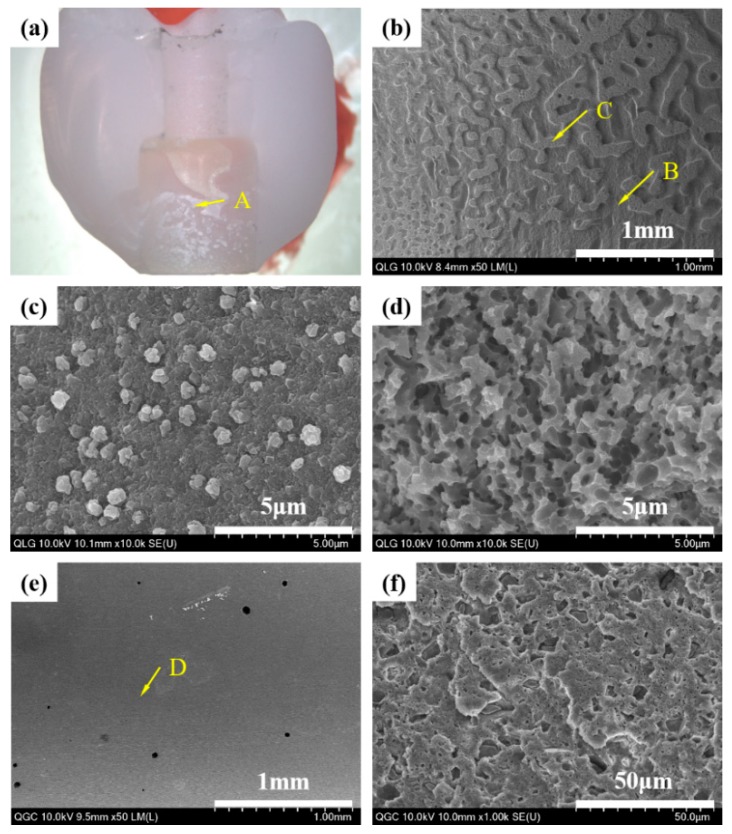
Photographic (**a**) and HR FE-SEM images of the lithium disilicate-reinforced glass ceramic crown that demonstrate the mode of failure; (**b**) the magnification of point A for the (milled, liner-bonded and glazed) MLG group: Inhomogeneous pattern of fracture surface; (**c**) the magnification of point B: Cohesive fracture occurred through the liner; (**d**) the magnification of point C: Adhesive fracture occurred at the interface with liner, and (**e**) magnification of point A for the milled, glazed, and cement-bonded (MGC) group: Homogeneous pattern of fracture surface; (**f**) the magnification of point D: Cement layer indicating adhesive fracture occurred at the interface with the zirconia ceramic abutment.

**Figure 10:**
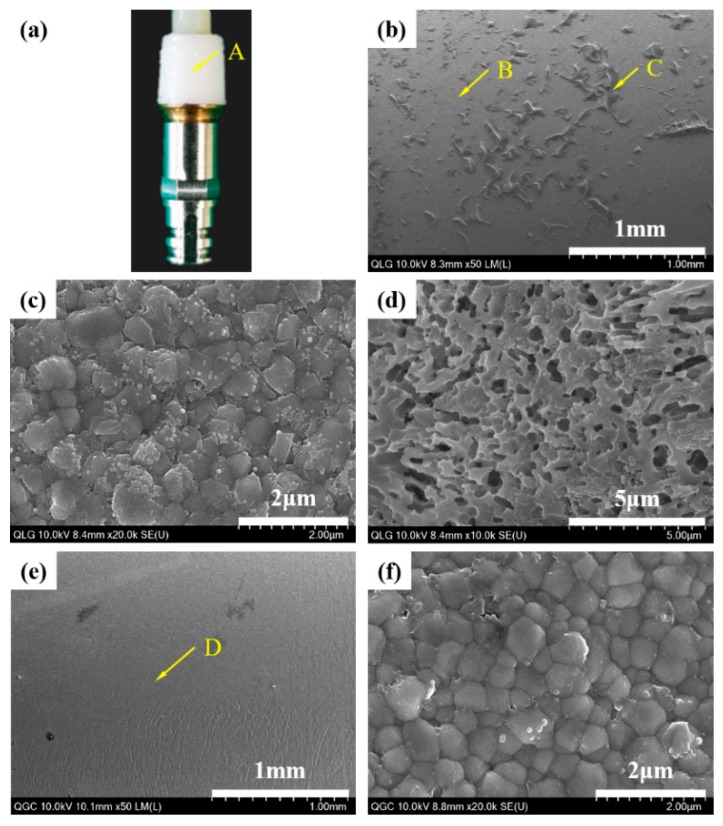
Photographic (**a**) and HR FE-SEM images (**b**–**d**) of the zirconia ceramic abutment; (**b**) the magnification of point A for the MLG group: Inhomogeneous pattern of fracture surface; (**c**) the magnification of point B: Adhesive fracture occurred at the interface with liner; (**d**) the magnification of point C: Cohesive fracture occurred through the liner; (**e**) the magnification of point A for the MGC group: Homogeneous pattern of fracture surface; (**f**) the magnification of point D: Zirconia crystals indicating that an adhesive fracture occurred at the interface with resin cement.

When the fractured surfaces of the lithium disilicate-reinforced glass ceramic crown and the zirconia ceramic abutment were visually evaluated with a high magnification images of HR FE-SEM, the mode of failure of the MLG group was mixed with adhesive and cohesive fractures propagated through the liner (Figure 9 and Figure 10b–d). However, the mode of failure was adhesive in the MGC group, and the fractures consistently occurred at the interface between the cement layer and the zirconia ceramic abutment (Figure 9 and Figure 10e,f).

## 4. Discussion

The liner-bonded ceramic crowns were superior to the cement-bonded ceramic crowns in developing a higher resistance to fracture. The bond strength of the lithium disilicate-reinforced glass ceramic to the zirconia ceramic was higher when the bonding was established with the lithium-disilicate liner than with an adhesive cement. The mean values of microtensile bond strength for liner bond and cement bond were 19.6 and 47.7 MPa, respectively, in this study. As an ideal biomaterial would have bonding forces included in the interval of 5–50 MPa, even if these values are mostly theoretical, both the liner and cement bond tested in this study exhibited bond strength within these limits [30]. The liner-bonded ceramic crowns resisted a higher loading without demonstrating a chipping than the cement-bonded crowns, although no significant difference was found in the fracture strength between the liner-bonded group and the cement-bonded-group. As confirmed in Figure 3 and Figure 5, the strong chemical bonding between the lithium disilicate liner and the zirconia ceramic was induced in the liner-bonded group, but there were lots of pores in the cement and mircro-gaps at the interface of the lithium disilicate-reinforced glass ceramic and cement in the cement-bonded group. Thus, it can be explained that the lower chipping strengths of the cement-bonded crowns than those of the liner-bonded crowns resulted from the stress concentration at defects of the interface when compression load was applied to the crown [31].

The bonding of ceramic restorations commonly involves acid etching and/or sandblasting procedures [32,33]. In the previous studies, it was reported that the shear bond strength of pre-sintered zirconia and the veneering porcelain did not change by conditioner treatment, but the failure mode was improved after thermal cycling [32]. It was also identified that the sandblasted zirconia surfaces had significantly higher shear bond strengths than non-treated and chemically etched surfaces without thermocycling, irrespective of conditioner treatment, and the highest shear bond strength and improved failure mode was confirmed by the application of both conditioner treatment and sandblasting [33]. Zirconia ceramics do not demonstrate the typical undercut features created by acid etching since they have chemical stability and almost no glass matrix. The silane treatment is not as effective as in silicate ceramics. The zirconia ceramics are usually roughened using an air-borne particle abrasion technology [34,35]. This procedure exposes sharp asperities of surface structures and increases surface area for bonding [36,37]. The resin cement should wet the surface and adhere to the irregular surface of ceramic restoration. This type of bonding relies on the surface characteristics of ceramic restorations, as well as the wettability and chemical compositions of resin cements, including the mechanisms of polymerization. According to Dérand et al. and Lüthy et al., the implant-supported zirconia restorations can fail at the interface when bonding is established with resin cements containing only 2,2-bis[p-(2′-hydroxy-3′-methacryloxypropoxy)phenylene]propane (bis-GMA) or 1,6-bis(methacryloxy-2-ethoxycarbonylamino)-2,4,4-trimethylhexane (UDMA) monomer [38,39]. In another study, it was identified that the shear bond strength of resin cements containing MDP of a zirconia ceramic was significantly improved by the application of a zirconia primer on both polished and blasted zirconia surfaces when comparing the results of the non-treatment of zirconia primer [40].

The one-piece system of zirconia abutments was found to present with some degree of misfit at the implant–abutment interface because of the challenge of the manufacturing process of the ceramic abutment [41,42]. Under loading, these abutments can induce an abrasion with micromotion at the implant–abutment interface [43]. The abutment screw can be loosened and lead to a bacterial colonization at the interface [6,44], as well a fracture failure of the implant-supported restoration. 

The two-piece system of zirconia abutments involves using a titanium base to establish an interfacial connection with similar materials to the implant to avoid possible complications resulting from the use of dissimilar materials [18,22]. These abutments were found to demonstrate a higher bond strength when the abutment was roughened with 110 µm alumina particles at 2.5 atm, coated with a ceramic primer, and bonded with a dual-cure self-adhesive resin cement [45]. However, the increased bond strength was possible when the titanium base was included when roughening the zirconia core by sandblasting. The bonded interface was intimate without demonstrating a gap between the abutment and cement layer. According to Ebert et al. [46], the retention of cement-bonded zirconia abutments was significantly higher when the bonded interfacial gap was less than 30 µm. 

The fracture resistance was higher when the zirconia abutment was designed to receive the support of the titanium base [17]. Gehrke et al. [7] used CAD/CAM technology to design zirconia abutments connected to an internal-hex titanium implant. The test specimens were thermocycled and cyclic loaded to fracture. They found that the two-piece system sustained a significantly higher fracture load than the one-piece system. Though the axial force generated during chewing typically does not exceed 220 N [47], the threshold for failure is estimated to be approximately 400 N according to the research conducted by Andersson et al. [48] and Att et al. [44].

Sandblasting is a common method of surface treatment used to bond zirconia ceramic restorations. The effect of this mechanical method, however, is limited to creating desired undercut features and increasing bond strength because of the dense polycrystalline structure of the zirconia ceramic [49,50]. However, the heat-bonding with the lithium disilicate liner was found to induce a chemical bonding to the zirconia ceramic, as indicated by the alteration of the crystal structure of zirconia at the interface of bonding. The zone of the chemical interaction with bonding revealed components of Si and Zr oxides which agreed with the previous research conducted by Jang et al. [31] and Aboushelib et al. [51]. 

The mode of failure was mixed with cohesive fracture propagated through the liner in the MLG group, whereas the failure was adhesive at the bonded interface of the ceramic and resin cement in the MGC group. The cement layer was thin at the interface and contained numerous pores and gaps. Meanwhile, no gap or pore was noted at the interface of the heat-bonding with the liner. These characteristics of bonding and mode of failure might have played a critical role in developing a higher fracture resistance of the implant-supported glass ceramic crown combined with a two-piece system of a zirconia abutment produced by a CAD/CAM procedure. However, a clinical study is yet to be conducted to determine the clinical success of the glass ceramic crown bonded to the zirconia abutment by means of heat-bonding with the liner.

## 5. Conclusions

The bond strength and fracture resistance of a milled lithium disilicate-reinforced glass ceramic bonded to a milled zirconia ceramic are affected by the modification of bonding procedures. The bond strength and fracture resistance were significantly higher when the bonding was established by means of heat-bonding with the lithium-disilicate liner than by a resin cement. The pattern of interfacial failure for the liner-bonded group was mixed with the fracture propagated through the liner. However, after visual inspection, the cement-bonded group demonstrated an adhesive failure at the interface of bonding. The results of this study suggest that heat-bonding with the liner can be an alternative to bond CAD/CAM-produced glass ceramic crowns to zirconia ceramic abutments in order to reduce the risk of crown dislodgement and fracture.

## Figures and Tables

**Table 1:** Weibull analysis data of a microtensile bond strength test for lithium disilicate-reinforced glass ceramic and zirconia of the liner-bonded group and the cement-bonded group.

	Group	Liner-Bonded	Resin Cement-Bonded
Parameter			
m		5.836	4.133
σ_o_		50.8	2.36
r^2^		0.961	0.958
σ_f(mean)_ ± SD		47.7 ± 8.7	19.6 ± 4.7
σ_f(min/med/max)_		32.8/45.5/58.7	10.9/20.6/26.0
N		12	12

where m = Weibull modulus; σ_o_ = characteristic strength in MPa; r^2^ = Weibull distribution regression coefficient squared; σ_f(mean)_ = mean fracture strength in MPa; σ_f(min/med/max)_ = minimum, median and maximum fracture strength in MPa; and N=number of samples.
